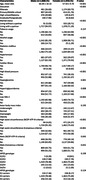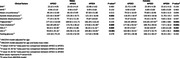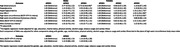# Sex‐specific differences in the association between ApoE genotypes and metabolic syndrome among middle‐aged and older rural Indians

**DOI:** 10.1002/alz.092699

**Published:** 2025-01-09

**Authors:** Shilna Azhuvalappil, Pravin Sahadevan, Jonas S Sundarakumar, Raghav Prasad

**Affiliations:** ^1^ Centre for Brain Research, Indian Institute of Science, Bangalore, Karnataka India

## Abstract

**Background:**

Metabolic Syndrome (MetS) is a significant health concern, characterized by a combination of cardio‐ vascular risk factors, influenced by genetic factors including the apolipoprotein E (ApoE). This study examines the sex‐ specific association between ApoE genotypes and MetS in a rural Southern Indian population

**Method:**

This cross‐ sectional study included 3741 participants aged ≥ 45 years from the rural Srinivaspura Aging, Neuro Senescence and COGnition (SANSCOG) cohort in Karnataka, India. All participants were measured for blood pressure, anthropometric measurements, and fasting concentrations of glucose, triglycerides, cholesterol, high‐density lipoprotein cholesterol, and low‐density lipoprotein cholesterol. ApoE Genotypes of 1808 males and 1933 females were included in this study. Based on ApoE genotype, participants were categorized into three distinct groups: E2 (ɛ2/ɛ2 or ɛ2/ɛ3 genotype), E3 (ɛ3/ɛ3 genotype) and E4 (ɛ4/ɛ4 or ɛ3/ɛ4 genotype) carriers. Using the data from baseline clinical and biochemical assessments, we defined MetS using two established criteria: National Cholesterol Education Program – Adult Treatment Panel III (NCEP‐ ATP III) and Consensus. Multivariable logistic regression models adjusted for demographic and lifestyle factors, were used to find the sex specific association between ApoE genotypes and MetS also with its individual components.

**Result:**

The study found a significant association between the ApoE E4 allele and MetS in females with an odds ratio (OR) of 1.31 (95% CI: 1.02, 1.69), as per the NCEP ATP III criteria. In contrast, no such association was found in males. We found females with E4 genotype also had a 1.33‐fold increase for hypertriglyceridemia (95% CI: 1.02, 1.73) and a 1.43‐fold increase in the odds of low HDL (95% CI: 1.03, 1.99). Interestingly, E4 female individuals had a 0.72‐ fold decrease in the odds of increased waist circumference (95% CI: 0.53, 0.99). In males, E4 carriers showed a 0.70‐ fold decreased odds of elevated blood pressure (95% CI: 0.54, 0.95). No significant association was found using the Consensus criteria between ApoE genotypes and MetS.

**Conclusion:**

This study highlights a significant sex‐ specific association between ApoE E4 allele and MetS and its components, particularly in females, in rural southern India.

**Keywords**: ApoE genotypes, Metabolic syndrome, sex specific